# Association of *TCF7L2* rs7903146 (C/T) Polymorphism with Type 2 Diabetes Mellitus in a Chinese Population: Clinical Characteristics and Ethnic Context

**DOI:** 10.3390/diagnostics15162110

**Published:** 2025-08-21

**Authors:** Yung-Chuan Lu, Teng-Hung Yu, Chin-Feng Hsuan, Chia-Chang Hsu, Wei-Chin Hung, Chao-Ping Wang, Wei-Hua Tang, Min-Chih Cheng, Fu-Mei Chung, Yau-Jiunn Lee, Thung-Lip Lee

**Affiliations:** 1 Division of Endocrinology and Metabolism, Department of Internal Medicine, E-Da Hospital, I-Shou University, Kaohsiung 82445, Taiwan; gregory.yclu@msa.hinet.net; 2 Division of Cardiology, Department of Internal Medicine, E-Da Hospital, I-Shou University, Kaohsiung 82445, Taiwan; tenghungy@gmail.com (T.-H.Y.); calvin.hsuan@msa.hinet.net (C.-F.H.); p7411906@ms19.hinet.net (W.-C.H.); ed100232@livemail.tw (C.-P.W.); chungfumei@gmail.com (F.-M.C.); 3 School of Medicine, College of Medicine, I-Shou University, Kaohsiung 82445, Taiwan; 4 Division of Cardiology, Department of Internal Medicine, E-Da Dachang Hospital, I-Shou University, Kaohsiung 80706, Taiwan; 5 Division of Gastroenterology and Hepatology, Department of Internal Medicine, E-Da Hospital, I-Shou University, Kaohsiung 82445, Taiwan; aladarhsu1107@gmail.com; 6 Health Examination Center, E-Da Dachang Hospital, I-Shou University, Kaohsiung 80706, Taiwan; 7 The School of Chinese Medicine for Post Baccalaureate, College of Medicine, I-Shou University, Kaohsiung 82445, Taiwan; 8 School of Medicine for International Students, College of Medicine, I-Shou University, Kaohsiung 82445, Taiwan; 9 Division of Cardiology, Department of Internal Medicine, Ministry of Health and Welfare Yuli Hospital, Hualien 98142, Taiwan; africapaul12@yahoo.com; 10 Faculty of Medicine, School of Medicine, National Yang Ming Chiao Tung University, Taipei 11230, Taiwan; 11 Department of Psychiatry, Taipei Veterans General Hospital, Yuli Branch, Hualien 98142, Taiwan; cmc@mail.vhyl.gov.tw; 12 Lee’s Endocrinologic Clinic, Pingtung 90000, Taiwan; lee@leesclinic.org

**Keywords:** *transcription factor 7-like 2* rs7903146, type 2 diabetes mellitus, gene polymorphism, inflammation, Chinese population

## Abstract

**Background/Objectives**: The *transcription factor 7-like 2* (*TCF7L2*) rs7903146 polymorphism has been strongly associated with type 2 diabetes mellitus (T2DM) in various populations; however, its impact on different ethnic groups is not fully understood. Given the distinct minor allele frequency in Chinese populations, this study aimed to analyze the association of rs7903146 with the risk of T2DM in a Han Chinese cohort and its relationship with relevant clinical parameters. **Methods**: We conducted a case–control study including 600 patients with type 2 diabetes mellitus (T2DM) and 511 sex-matched non-diabetic controls of Han Chinese descent. The *TCF7L2* rs7903146 (C/T) polymorphism was genotyped using a TaqMan™ SNP assay. Clinical parameters, including body mass index (BMI), fasting plasma glucose, hemoglobin A1c, lipid profile, and high-sensitivity C-reactive protein (hs-CRP), were compared between genotypes. Logistic regression analyses were performed under a dominant genetic model (CT/TT vs. CC), adjusting for age, sex, systolic and diastolic blood pressure, BMI, and smoking status. Subgroup analyses were conducted by sex, BMI category, age at diagnosis, and family history of T2DM. Given the exploratory nature of this study and the low frequency of the TT genotype, no formal correction for multiple testing was applied. **Results**: Frequencies of the CT and TT genotypes were higher in the diabetic group (*p* = 0.045) and were significantly associated with an increased risk of T2DM under a dominant genetic model (adjusted OR = 2.24, *p* = 0.025). Individuals with CT/TT genotypes had elevated fasting glucose and hs-CRP levels; these genotypes were also linked to higher BMI in the female T2DM patients. The T allele frequency varied across ethnic groups, being lowest in East Asians and highest in Latin (Brazilian/mixed ancestry) populations. Mechanistically, the T allele may contribute to T2DM via altered *TCF7L2* expression, impaired insulin secretion, inflammation, and metabolic dysregulation. **Conclusions**: The *TCF7L2* rs7903146 T allele was associated with an increased risk of T2DM and higher fasting glucose and hs-CRP levels in this Han Chinese cohort. The CT/TT genotypes were also associated with higher BMI in the female T2DM patients. While the findings are consistent with the known effects of this variant in other populations, mechanistic hypotheses such as the involvement of inflammatory or metabolic pathways remain hypothetical and warrant further functional validation.

## 1. Introduction

Type 2 diabetes mellitus (T2DM) is a complex and multifactorial metabolic disorder characterized by chronic hyperglycemia, resulting from impaired insulin secretion and/or insulin resistance [1,2]. It is a serious public health issue globally, with a rapidly increasing prevalence, particularly in developing countries [3]. In Taiwan, the prevalence of T2DM has reached epidemic proportions, driven by lifestyle changes, urbanization, and genetic predisposition [4]. Both environmental and genetic factors contribute to the pathogenesis of T2DM [5], and advances in genetic research have shed light on the role of specific genetic polymorphisms in disease susceptibility [6].

Among the genetic factors implicated in T2DM, the *transcription factor 7-like 2* (*TCF7L2*) gene has garnered significant attention [7,8,9]. *TCF7L2* is located on chromosome 10q25.2 and encodes a transcription factor involved in the Wnt signaling pathway, which plays a pivotal role in pancreatic beta-cell function, insulin secretion, and glucose metabolism [10,11]. Numerous genome-wide association studies have reported that rs7903146 polymorphism in the *TCF7L2* gene is a strong genetic risk factor for T2DM across diverse populations [7,8,9]. The T allele is thought to alter *TCF7L2* expression and disrupt glucose metabolism [12] and may also be linked to metabolic traits such as chronic inflammation and increased adiposity, further contributing to disease progression.

Although the association between the *TCF7L2* rs7903146 polymorphism and T2DM has been well established in several populations, results in East Asians have been inconsistent, likely due to the low frequency of the T allele. In addition, few studies have explored the potential influence of this variant on clinical and metabolic traits or its interaction with patient-specific factors such as sex, body mass index (BMI), or age at disease onset. In this study, we aimed to investigate the association between the rs7903146 (C/T) variant and risk of T2DM in a Han Chinese population, as well as its relationship with relevant clinical parameters. We also examined genotype distribution across various subgroups and compared allele frequencies with those reported in other ethnic populations. These findings may provide further insights into how this common genetic variant contributes to the heterogeneity of T2DM presentation across different populations.

## 2. Materials and Methods

### 2.1. Participants

The study participants were patients with T2DM who attended our diabetes clinic between November 2023 and November 2024 and participated in a disease management program. T2DM was diagnosed following the criteria reported by the American Diabetes Association [13]. We excluded patients with signs indicative of type 1 diabetes, such as the continuous need for insulin within 1 year of the diagnosis, acute onset with heavy ketonuria (3+), or diabetic ketoacidosis. We also enrolled a sex-matched control group, which included individuals attending routine check-up visits who had no clinical evidence of coronary artery, renal, or cerebrovascular disease and no clinical evidence of T2DM, defined as no history or family history of diabetes, and fasting plasma glucose levels below 100 mg/dL. Detailed personal and family disease histories were collected from all participants by trained personnel, and questionnaires were carefully reviewed for completeness and accuracy. A family history of T2DM was defined as having at least one immediate family member (father, mother, sibling, or child) diagnosed with T2DM. All of the participants gave written informed consent to participate in this case–control study, which was approved by the Human Research Ethics Committee of E-Da Hospital.

### 2.2. Measurements

All of the enrolled participants resided in the same region during the study period and were of Han Chinese descent. Each participant underwent a comprehensive physical examination, routine blood and urine biochemical analyses, and were evaluated for the occurrence and severity of micro- and macrovascular complications. Height (to the nearest 0.5 cm) and weight (to the nearest 0.1 kg) were measured using standard procedures, and the BMI was calculated. Waist circumference was measured at the narrowest point between the uppermost lateral border of the right iliac crest and the lowest rib, while hip circumference was measured at the widest point, both with an accuracy of 0.1 cm. Each measurement was taken twice, and the waist-to-hip ratio (WHR) was calculated. Obesity was defined as BMI ≥ 27 kg/m^2^, according to the criteria of the Department of Health in Taiwan. Blood pressure readings (both systolic blood pressure [SBP] and diastolic blood pressure [DBP]) were taken by a trained nurse using a digital device (Omron HEM-907, Kyoto, Japan). Measurements were taken while the participants were seated, after a 5 min rest. Plasma biochemical parameters were measured after overnight fasting, including glucose, creatinine, uric acid, triglycerides, total cholesterol, and low- and high-density lipoprotein cholesterol (LDL-C/HDL-C), using a biochemical analyzer (Hitachi 7170A, Tokyo, Japan), following protocols detailed in prior studies [14]. Hemoglobin A1c (HbA1c) levels were measured using an automated analyzer (HLC-723G8; Tosoh Corporation, Tokyo, Japan). Serum creatinine levels were determined using the Jaffe method. Plasma high-sensitivity C-reactive protein (hs-CRP) levels were measured using an immunochemistry device (Beckman Coulter IMMAGE, Beckman Coulter, Brea, CA, USA). The assay had a detection limit of 0.2 mg/L, and each sample was analyzed in duplicate to ensure accuracy. Participants who had smoked within one year prior to the examination were classified as current smokers, while those who had quit smoking more than one year before the examination were classified as non-smokers.

### 2.3. Selection and Determination of the TCF7L2 Genotype (rs7903146)

The rs7903146 (C/T) polymorphism in the *TCF7L2* gene was selected for genotyping based on its consistent and strong association with T2DM across diverse populations, as reported in multiple large-scale genome-wide association studies and meta-analyses [7,8,9]. Although its regulatory role has already been well established in prior studies, our primary aim was to investigate the association of this well-characterized single nucleotide polymorphism (SNP) with T2DM and related metabolic traits in a Han Chinese population, in whom the minor allele frequency is low, and population-specific effects remain underexplored.

Genomic DNA was isolated from whole blood samples using the QIAamp DNA Blood Mini Kit (Qiagen, Hilden, Germany) and diluted in Tris/EDTA buffer (10 mM Tris, 1 mM EDTA, pH 7.8). DNA concentration was assessed by measuring absorbance at 260 nm, and samples were stored at −20 °C until PCR amplification. Genotyping of the rs7903146 SNP was performed using the QuantStudio 3 real-time PCR system (Thermo Fisher Scientific, Waltham, Massachusetts, United States) with the TaqMan™ SNP Genotyping Assay (Assay ID: C-29347861-10). The target context sequence was TAGAGAGCTAAGCACTTTTTAGATA[C/T]TATATAATTTAATTGCCGTATGAGG. PCR reactions were conducted in a 10 μL total volume consisting of 5 ng genomic DNA, 0.25 μL of 40× predesigned TaqMan™ SNP Genotyping Assay (containing two unlabeled primers and VIC^®^/FAM™ dye-labeled MGB probes), and 5 μL of 2× TaqMan^®^ GTXpress™ Master Mix. The thermal cycling protocol was initial denaturation at 95 °C for 2 min, followed by 40 cycles of 95 °C for 15 s and 60 °C for 40 s. Finally, genotype calls were determined based on allelic discrimination plots generated by QuantStudio™ 3 software using fluorescence signal intensity to distinguish among CC, CT, and TT genotypes.

### 2.4. Statistical Analysis

Data normality was determined using the Kolmogorov–Smirnov test. Normally distributed continuous data are shown as mean (SD), and non-normally distributed data as median (interquartile range). Due to skewed distribution, plasma triglyceride and hs-CRP values were logarithmically transformed before analysis. Statistical differences in continuous data were evaluated with either an unpaired Student’s *t*-test for normally distributed variables or a Mann–Whitney U test for skewed variables, as appropriate. Categorical variables are shown as count (percentage), and group comparisons were performed using the chi-square test or Fisher’s exact test. The chi-square test was also used to determine deviations in genotype frequencies from the Hardy–Weinberg equilibrium. Associations between the risk of T2DM and genotype frequencies were evaluated using logistic regression analysis, adjusting for age, sex, blood pressure, BMI, and smoking status. The results are given as odds ratios (ORs) and 95% confidence intervals (CIs). Given the exploratory nature of this study and the low frequency of the TT genotype, no formal correction for multiple testing was applied; all *p*-values are presented as unadjusted. A Pearson correlation heat map was also used to examine the relationships between *TGF7L2* genotypes and the incidence of T2DM.

As the TT genotype was only identified in a few subjects, we evaluated differences in variables between subjects with and without the T variant by comparing the CC genotype with the combined CT and TT genotypes. A post hoc power analysis was performed, and assuming a T allele frequency of approximately 4% in controls and 8% in cases (corresponding to an OR of 2.0), this study had approximately 81% power to detect a significant association at a two-sided significance level of 0.05. Subgroup analyses were conducted among the patients with diabetes based on sex, family history of T2DM (yes or no), BMI (<27 or ≥27 kg/m^2^), and age when diabetes was diagnosed (<60 or ≥60 years). Differences between genotype groups were assessed using an unpaired Student’s *t*-test or Wilcoxon rank-sum test, as appropriate. All statistical analyses were conducted using JMP software version 10.0 for Windows (SAS Institute, Cary, NC, USA), with a *p*-value < 0.05 considered statistically significant.

## 3. Results

### 3.1. Characteristics of the Control and Diabetic Groups

The patients with diabetes were older and had a significantly higher rate of current smoking, as well as higher BMI, WHR, SBP, serum triglycerides, fasting glucose, and HbA1c compared to the non-diabetic controls (Table 1). In addition, the patients with diabetes had lower serum total cholesterol, LDL-C, and HDL-C compared to the controls.

### 3.2. Association Between TCF7L2 Genotypes and T2DM

The distribution of *TCF7L2* C/T genotypes in control and diabetic groups is presented in Table 2 and was consistent with the Hardy–Weinberg equilibrium. The difference in overall genotype distribution between the T2DM and control groups was not significant (*p* = 0.114). However, as only a few subjects had the TT genotype, we compared those with and without the T variant by grouping CT and TT genotypes together and comparing them against the CC genotype. The analysis showed a significant difference in the distribution of *TCF7L2* CT and TT genotypes between the T2DM and control groups (*p* = 0.048). Higher frequencies of the TT and CT genotypes were observed in the T2DM group, indicating a potential association between *TCF7L2* gene polymorphisms and T2DM (CT and TT vs. CC: crude OR 1.86, 95% CI: 1.02–3.58, *p* = 0.045). Furthermore, to explore potential associations in specific subgroups of patients with diabetes, we conducted analyses stratified by BMI (≥27 or <27 kg/m^2^), sex, age at diabetes onset (≥60 or <60 years), and a family history of diabetes (yes or no). No significant associations were observed between the *TCF7L2* polymorphisms and T2DM in these analyses (Table 2). In addition, we analyzed and reported the effect sizes for relevant comparisons, which are now presented in the Online-Only Supplementary Data (See Table S1) and in Table 1. Table 3 presents the adjusted odds ratios (AORs) for the association between T2DM patients and controls under the dominant model and allele-based inheritance. The AORs for the C and T alleles were 0.20 (95% CI: 0.05–0.75, *p* = 0.016) and 2.22 (95% CI: 1.16–4.46, *p* = 0.016), respectively. For the dominant model, the AOR was 2.24 (95% CI: 1.10–4.80, *p* = 0.025).

### 3.3. Clinical and Biochemical Characteristics of All Subjects, the Diabetes and Control Groups, Stratified by TCF7L2 Genotype

The clinical and biochemical data of the diabetes and control groups, categorized by the *TCF7L2* genotype, are shown in Table 4. Individuals with the CT and TT genotypes had significantly elevated fasting glucose levels compared to those with the CC genotype among both the overall cohort and control group (*p* < 0.05). In addition, individuals with the CT and TT genotypes had significantly elevated hs-CRP levels compared to those with the CC genotype in both the overall cohort and T2DM patients (*p* < 0.05). Notably, female T2DM patients with the CT and TT genotypes also had a significantly higher BMI (*p* = 0.024).

### 3.4. Genotype Distribution of TCF7L2 rs7903146 (C/T) Polymorphism in Different Ethnic Groups

**Figure 1:**
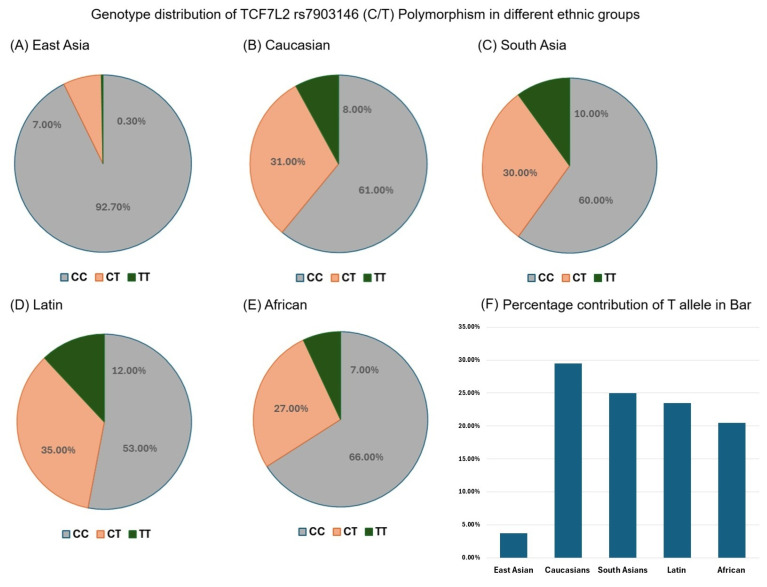
Genotype distribution of *transcription factor 7-like 2* (*TCF7L2*) rs7903146 (C/T) polymorphism in different ethnic groups. The pie charts show the genotype frequencies (CC, CT, and TT) of the *TCF7L2* rs7903146 polymorphism in (**A**) East Asian, (**B**) Caucasian, (**C**) South Asian, (**D**) Latin (Brazilian/mixed), and (**E**) African populations. (**F**) Bar chart illustrating the percentage contribution of the T allele of the *TCF7L2* rs7903146 polymorphism across these five ethnic groups. The proportion of each genotype in the pie chart is represented as a percentage of the total number of individuals within each ethnic group, and the data in the bar chart reflect the relative frequency of the T allele within each populations.

The genotype distribution of the *TCF7L2* rs7903146 (C/T) polymorphism across various ethnic groups was examined based on two published meta-analyses [7,15] and is presented in Figure 1. In the East Asian population, the CC genotype was predominant (92.7%), with relatively low frequencies of the CT (7.0%) and TT (0.3%) genotypes (Figure 1A). In contrast, the distribution was more balanced in the Caucasian population, with frequencies of 61.0%, 31.0%, and 8.0% for the CC, CT, and TT genotypes, respectively (Figure 1B). In South Asians, the frequencies of the CC, CT, and TT genotypes were 60.0%, 30.0%, and 10.0% (Figure 1C), whereas the prevalence of the T allele was highest in the Latin (Brazilian/mixed ancestry) population, with genotype frequencies of 53.0%, 35.0%, and 12.0% for CC, CT, and TT, respectively (Figure 1D). Among the African population, the CC genotype was most frequent (66.0%), followed by CT (27.0%) and TT (7.0%) (Figure 1E).

### 3.5. TCF7L2 rs7903146 T Allele Frequency in Different Ethnic Groups

Two previous meta-analyses found that the frequency of the T allele at the *TCF7L2* rs7903146 locus varied substantially among different ethnic groups (Figure 1), with the lowest frequency among individuals of East Asian descent (3.7%) and the highest among those of Latin (Brazilian/mixed) descent (29.5%), followed by South Asians (25.0%), Caucasians (23.5%), and Africans (20.5%) [7,15]. In our Han Taiwanese cohort, the CT and TT frequency was low (4.2%), similar to that observed in other East Asian populations. These findings indicate substantial ethnic differences in the distribution of the rs7903146 T allele, which may have implications for population-specific genetic susceptibility to T2DM.

## 4. Discussion

Our findings revealed a significantly higher prevalence of the *TCF7L2* CT and TT genotypes in the patients with T2DM compared to the controls. In addition, univariate and multivariate logistic regression analyses, adjusted for age, sex, SBP, DBP, BMI, and smoking, demonstrated independent associations between T2DM and the *TCF7L2* CT and TT genotypes, as well as the T allele. While previous epidemiological studies have highlighted the key role of environmental factors in the development of T2DM [16,17], not all individuals exposed to these factors develop the disease [18,19], and molecular epidemiological studies have suggested that both environmental and genetic factors contribute to T2DM susceptibility [20,21].

Previous studies have identified *TCF7L2* as one of the most robust genetic markers associated with the risk of T2DM across diverse populations [10,22,23]. A recent study conducted in India reported that the T allele of the rs7903146 (C/T) SNP was associated with a two-fold increased risk of T2DM, and that individuals with the heterozygous CT genotype had a 1.96-fold elevated risk [24]. However, in a study conducted in Ghana, Danquah et al. reported a *TCF7L2* rs7903146 T allele frequency comparable to that in Caucasians, although its association with T2DM was slightly weaker [25]. In contrast, Sun et al. reported that *TCF7L2* SNPs (rs7895340, rs11196205, rs7901695, rs7903146, and rs12255372) were not associated with T2DM in a cohort of Liannan Yao participants [26].

In the present study, we further analyzed the genotype distribution of the *TCF7L2* rs7903146 polymorphism across major ethnic groups based on two published meta-analyses [7,15]. The results revealed substantial differences among groups, with the T allele being most prevalent in Latin American (~30%) and South Asian (~25%) populations, moderate in Caucasian (~24%), and lowest in East Asian and African (~4–21%) populations. Notably, the CC genotype was predominant among East Asians (>80%), while the TT genotype was rare. These findings align with a previous study and emphasize the importance of considering ethnic background when evaluating genetic risk for T2DM [27]. Differences in allele frequency across populations may partly account for observed differences in disease prevalence and influence the predictive value of genetic risk models. Moreover, although rs7903146 has been studied in other ethnic groups, our results not only confirm its association with T2DM in a Han Chinese cohort but also extend prior findings by examining potential genotype–phenotype correlations in clinically meaningful subgroups, including BMI, sex, age at onset, and family history of diabetes. These analyses may suggest differential effects of the T allele in specific groups of patients and provide additional information for personalized risk assessment.

In our Han Taiwanese cohort, the CT and TT frequency was low (4.2%), similar to that observed in other East Asian populations. A significant association was found between the CT and TT genotypes and increased susceptibility to T2DM. The *TCF7L2* risk allele has been reported to confer up to a 50% increased risk of T2DM, with a population-attributable risk ranging from 10% to 25%, depending on allele frequency [28,29,30]. Moreover, several studies have shown that *TCF7L2* variants can predict diabetes risk in individuals with impaired glucose tolerance [10,31], consistent with the significantly elevated fasting glucose levels we observed in individuals carrying these genotypes. Nevertheless, the precise mechanisms underlying these associations remain to be fully elucidated.

The SNP rs7903146 is located on chromosome 10q25.3 in the fourth intron of the *TCF7L2* gene. *TCF7L2* encodes a transcription factor with a high-mobility group (HMG) box, which serves as a key effector of the canonical WNT signaling pathway involved in cell differentiation [32,33,34]. It regulates genes, including c-myc and cyclin D1, that mediate the cell cycle G1-S phase transition [33,34,35]. While its primary link to T2DM has been attributed to its role in insulin secretion, most studies have focused on its effects on the proliferation of beta cells in the pancreas, where altered *TCF7L2* expression leads to impaired insulin secretion [10,31,36]. Furthermore, a dominant-negative mutant of *TCF7L2* has been shown to abolish levels of pro-glucagon mRNA [37,38], thereby affecting glucagon-like peptide-1 (GLP-1) production and gluconeogenesis [39]. Taken together with our findings, *TCF7L2* gene polymorphisms may play an important role in heritable genetic–environmental interactions in T2DM. In addition, in an in vivo study, Chen et al. demonstrated that inactivating the *TCF7L2* gene in mature adipocytes by removing the HMG-box DNA-binding domain resulted in glucose intolerance throughout the body and hepatic insulin resistance, accompanied by an increase in subcutaneous adipose tissue, adipocyte hypertrophy, and inflammation [32]. In the present study, we found that individuals with the *TCF7L2* CT and TT genotypes had higher levels of hs-CRP compared to those with the CC genotype, both in the overall cohort and among the T2DM patients. These findings suggest that individuals carrying the CT and TT genotypes may be more prone to inflammation, potentially increasing their risk of developing T2DM. This elevated risk may be mediated through mechanisms involving greater subcutaneous fat accumulation, adipocyte hypertrophy, and inflammation processes known to be influenced by altered *TCF7L2* function.

**Figure 2:**
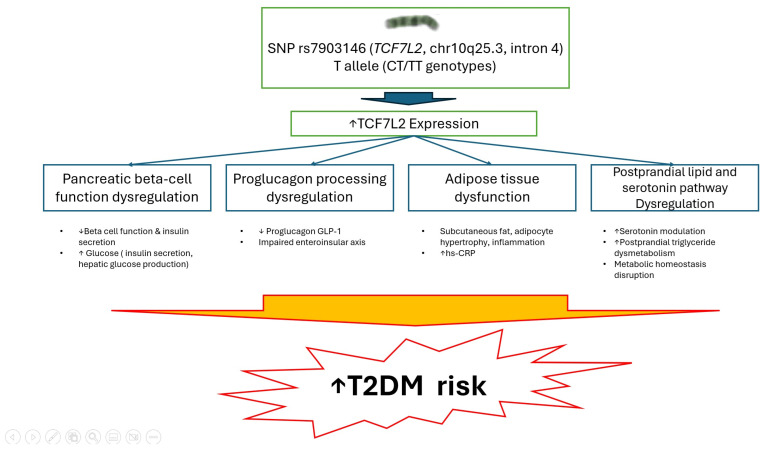
Hypothetical mechanisms linking the T allele of *transcription factor 7-like 2* (*TCF7L2*) rs7903146 to increased type 2 diabetes risk. The T allele (CT/TT genotypes) of the rs7903146 variant in the *TCF7L2* gene is associated with increased *TCF7L2* expression, which might contribute to multiple metabolic dysfunctions, including pancreatic β-cell dysfunction and impaired insulin secretion; dysregulation of proglucagon processing and glucagon-like peptide-1 production; adipose tissue inflammation and hypertrophy; and alterations in postprandial lipid and serotonin metabolism. These disruptions might collectively impair metabolic homeostasis and contribute to an elevated risk of type 2 diabetes mellitus. References supporting each hypothetical mechanism are indicated in brackets.

The elevated risk of T2DM associated with *TCF7L2* variants has been linked to dysfunction in the enteroinsular axis, increased gene expression in pancreatic islets, and impaired insulin secretion [10]. The results of both non-adjusted and adjusted analyses in the present study showed that the *TCF7L2* CT and TT genotypes were significantly associated with T2DM (Table 2 and Table 3). Similarly, Kumar et al. [24], Cropano et al. [40], and Ding et al. [15] reported a significant association between *TCF7L2* rs7903146 and T2DM. In addition, Leiherer et al. reported a significant association between serotonin and the rs7903146 genotype in an analysis adjusting for T2DM and other common metabolites. Given that serotonin is known to play roles in metabolic homeostasis and T2DM, these findings suggest that the well-established influence of *TCF7L2* polymorphisms on the risk of T2DM may, at least in part, involve a serotonin-dependent pathway [41]. The mechanism underlying the link between the rs7903146 risk CT and TT genotypes and T2DM could be explained by three key pathways. First, the T allele of *TCF7L2* rs7903146 has been shown to impair beta cell function, lowering the disposition index and proinsulin secretory efficiency. It has also been shown to decrease the ability of insulin to suppress hepatic glucose production, thereby contributing to hyperglycemia [40]. Variations in the *TCF7L2* gene disrupt the transcriptional regulation of target genes, leading to beta cell dysfunction, a key contributor to the development of T2DM. Second, postprandial triglyceride dysmetabolism has been associated with rs7903146 risk alleles and *TCF7L2* expression in adipocytes. In a study of glucose-intolerant Asian Indians, *TCF7L2* expression in visceral adipose tissue was shown to be upregulated and correlated with postprandial triglycerides and glycemia. The *TCF7L2*-mediated modulation of postprandial triglyceride metabolism may contribute to the risk of diabetes [42]. Third, the *TCF7L2* gene encodes a transcription factor containing an HMG box that has been linked to blood glucose homeostasis. Its function is believed to involve regulating proglucagon gene expression in enteroendocrine cells through the Wnt signaling pathway [23]. In addition, a study by Lyssenko et al. highlighted the impact of the T allele of rs7903146 in *TCF7L2* on the enteroinsular axis, as well as its influence on the relationship between the incretin hormone gastric inhibitory polypeptide and its target hormones, glucagon and insulin [10]. They also observed that increased *TCF7L2* expression associated with the T allele in human islets in vitro was associated with impaired insulin secretion both in vivo and in vitro, and that this contributed to the risk of T2DM (Figure 2).

An interesting finding in this study is that the female T2DM patients with the CT and TT genotypes had a significantly higher BMI compared to their counterparts with the CC genotype. This finding highlights a potential sex-specific interaction between *TCF7L2* variants and metabolic factors. Previous studies have suggested that hormonal differences, particularly estrogen, may modulate the impact of *TCF7L2* variants on glucose and lipid metabolism [43]. Estrogen receptors are known to interact with components of the Wnt signaling pathway, which may explain the observed genotype-specific effects on BMI in the female patients. This highlights the need for further research into sex-specific genetic and hormonal interactions to better understand the complex mechanisms underlying T2DM.

In the subgroup analyses of the T2DM group based on sex, obesity status, age when diabetes was diagnosed, and family history of diabetes, no significant associations were found between the rs7903146 polymorphism and T2DM. While *TCF7L2* is widely recognized as one of the strongest genetic risk factors for T2DM, its impact may be influenced by population-specific characteristics, environmental factors, or interactions with other genetic variants. In contrast to our findings, previous studies have suggested that the effect of rs7903146 may vary depending on specific subgroups, such as obesity status [40] or family history [44]. The lack of significant findings in our subgroup analysis could be attributed to sample size limitations, genetic heterogeneity, or differences in lifestyle and metabolic profiles among the study population. Future studies with larger sample sizes and well-characterized subgroups may help clarify whether rs7903146 plays a more nuanced role in the susceptibility to diabetes across different patient populations. In addition, investigating genetic–environmental interactions may provide further insights into the complex genetic architecture of T2DM.

Finally, Figure 2 illustrates the hypothetical mechanisms by which the rs7903146 T allele might increase the risk of T2DM. Based primarily on previously published studies rather than direct experimental evidence from the present work, carriers of the T allele (CT/TT genotypes) have been reported to exhibit increased *TCF7L2* expression, which could impair pancreatic beta-cell function, reduce GLP-1 production, and lead to dysregulated insulin secretion. The T allele has also been suggested to be associated with adipose tissue dysfunction, elevated hs-CRP levels, and abnormalities in lipid and serotonin metabolism, all of which may potentially contribute to disrupted metabolic homeostasis. Given the cross-sectional and observational design of our study, these mechanistic interpretations should be regarded as speculative, and future functional studies are warranted to verify or refute these proposed pathways.

### Study Limitations

Despite the significant findings in this study, there are also several limitations. First, we could not examine causal relationships between *TCF7L2* genotypes and T2DM or related metabolic parameters due to the cross-sectional study design. While we observed strong associations, longitudinal studies are required to verify our findings and establish whether *TCF7L2* variants contribute to the development and progression of T2DM over time. Second, our study focused on a limited number of metabolic and inflammatory markers, such as fasting glucose and hs-CRP. Expanding the analysis to include biomarkers such as lipid profile, insulin level, and adipokines could provide further insights into the mechanisms linking *TCF7L2* variants to T2DM. Third, this study lacks functional investigations to directly clarify the molecular mechanisms behind the observed associations. Although our results imply that *TCF7L2* variants affect glucose metabolism and inflammation, further in vivo and in vitro studies are needed to validate these pathways and explore potential therapeutic targets. Fourth, the numbers of patients in the subgroup analyses may have been insufficient to detect small but significant effects¸ especially for the TT genotype. Further studies with a larger number of patients are warranted to verify our results. Fifth, our study did not assess epigenetic mechanisms, such as DNA methylation or histone modifications, at the *TCF7L2* locus [45]. These epigenomic factors regulate gene expression and may influence the functional impact of rs7903146 in a tissue-specific manner, particularly in pancreatic islets and adipose tissue [46]. Finally, despite adjusting for several key covariates, the possibility of residual confounding cannot be excluded. In addition, the observational nature of our study limits causal inference, underscoring the need for future validation using functional genomics, gene expression analyses, or Mendelian randomization approaches to better establish causality and biological relevance. Investigating these mechanisms in the future may help clarify how genetic and epigenetic interactions jointly contribute to disease susceptibility and clinical heterogeneity in T2DM. Addressing these limitations in future research will enhance the understanding of the complex interplay between genetic and environmental factors in T2DM and improve the translation of genetic insights into clinical practice.

## 5. Conclusions

Our results showed that the *TCF7L2* rs7903146 T allele was associated with an increased risk of T2DM in this Han Chinese population, consistent with findings in other ethnic groups. While the overall frequency of the T allele is lower in East Asians, the carriers of the CT and TT genotypes in this study had higher fasting glucose and hs-CRP levels, and the female patients had higher BMI. These results validate previous genetic associations in this ethnic context and highlight potential genotype–phenotype relationships that may inform population-specific risk stratification and personalized interventions. Our findings also underscore the importance of considering ethnic allele frequency and metabolic profiles in the interpretation of the genetics of diabetes.

## Figures and Tables

**Table 1:** Clinical and biochemical characteristics of the study groups.

Parameters	Type 2 Diabetes	Non-Diabetic Controls	*p*-Value	Effect Sizes
N	600	511		
Sex (% men)	49.2	50.5	0.660	0.95
Age (year)	73.3 ± 6.5 (72.7–73.8)	46.9 ± 12 (25.9–47.9)	<0.0001	
Duration of disease (year)	15.8 ± 8.1 (15.1–16.4)	-	-	
BMI (kg/m^2^)	25.9 ± 3.7 (25.6–26.2)	24.0 ± 4.0 (23.7–24.4)	<0.0001	
Age at diagnosis (year)	54.6 ± 9.5 (53.8–55.3)	-	-	
WHR Men	0.95 ± 0.07 (0.94–0.96)	0.89 ± 0.06 (0.88–0.90)	<0.0001	
Women	0.89 ± 0.06 (0.88–0.90)	0.79 ± 0.06 (0.78–0.80)	<0.0001	
Systolic BP (mm Hg)	134 ± 18 (132–135)	125 ± 18 (123–126)	<0.0001	
Diastolic BP (mm Hg)	78 ± 11 (77–29)	77 ± 12 (76–78)	0.213	
Total cholesterol (mg/dL)	192.8 ± 40.0 (189.6–196.1)	208.2 ± 39.2 (204.7–211.6)	<0.0001	
Triglyceride (mg/dL)	122.0 (85.3–168.0)	92.0 (63.0–138.0)	<0.0001 *	
HDL cholesterol (mg/dL)	50.9 ± 13.3 (49.8–52.0)	61.1 ± 19.0 (59.5–62.8)	<0.0001	
LDL cholesterol (mg/dL)	108.7 ± 35.1 (105.9–111.6)	131.1 ± 36.1 (127.9–134.3)	<0.0001	
Fasting glucose (mg/dL)	151.5 ± 56.5 (147.0–156.1)	91.0 ± 8.4 (90.2–91.7)	<0.0001	
HbA1c (%)	8.3 ± 2.0 (8.2–8.5)	5.5 ± 0.4 (5.4–5.5)	<0.0001	
Treatment (%)				
Oral drugs	87.4	-	-	
Insulin	5.4	-	-	
Oral drugs + insulin	7.2	-	-	
Antihypertensive therapy (%)	28.0	-	-	
Statin therapy (%)	57.5	-	-	
Current smoker (%)	27.8	17.2	<0.0001	1.85

Data are presented as mean ± standard deviation (95% confidence interval) for normally distributed variables, median (interquartile range) for skewed variables, and percentage for categorical variables. *p*-values are uncorrected and calculated using an independent *t*-test for normally distributed variables, the Mann–Whitney U test for skewed variables, and a χ^2^ test for categorical variables. Abbreviations: BMI—body mass index; WHR—waist-to-hip ratio; BP—blood pressure; HDL—high-density lipoprotein cholesterol; LDL—low-density lipoprotein cholesterol. * Indicates significance based on analysis of log-transformed variables.

**Table 2:** *Transcription factor 7-like 2* (rs7903146) genotype distributions in the two study groups.

Genotype	Controls (*n*, %)	T2DM (*n*, %)	Type 2 Diabetes Mellitus							
			WFH (*n*, %)	WOFH ^a^ (*n*, %)	ADO ≥ 60 (*n*, %)	ADO < 60 (*n*, %)	BMI ≥ 27 kg/m^2^ (*n*, %)	BMI < 27 kg/m^2^ (*n*, %)	Male (*n*, %)	Female (*n*, %)
N (%)	511	600	224 (62.2)	136 (37.8)	180 (30.0)	420 (70.0)	203 (33.8)	397 (66.2)	295 (49.2)	305 (50.8)
CC	496 (97.1)	568 (94.7)	208 (92.9)	128 (94.1)	173 (96.1)	395 (94.1)	194 (95.6)	374 (94.2)	280 (94.9)	288 (94.4)
CT	15 (2.9)	31 (5.2)	15 (6.7)	8 (5.9)	7 (3.9)	24 (5.7)	9 (4.4)	22 (5.5)	15 (5.1)	16 (5.3)
TT	0 (0.0)	1 (0.2)	1 (0.5)	0 (0.0)	0 (0.0)	1 (0.2)	0 (0.0)	1 (0.3)	0 (0.0)	1 (0.3)
CT + TT	15 (2.9)	32 (5.3) *	16 (7.1)	8 (5.9)	7 (3.9)	25 (6.0)	9 (4.4)	23 (5.8)	15 (5.1)	17 (5.6)
Crude OR ^b^	-	1.86	1.23	0.81	0.64	1.56	0.75	1.33	0.91	1.10
(95% CI)	-	(1.02–3.58)	(0.53–3.11)	(0.32–1.90)	(0.25–1.43)	(0.70–3.98)	(0.33–1.61)	(0.62–3.08)	(0.44–1.86)	(0.54–2.28)
*p*-value	-	0.045	0.639	0.639	0.289	0.289	0.477	0.477	0.790	0.790

Abbreviations: WFH—with a family history of diabetes; WOFH—without a family history of diabetes; ADO—age at diabetes onset; BMI—body mass index; CI—confidence interval. ^a^: 240 participants lacking family history data were excluded from subgroup analysis. ^b^: Logistic regression was used to assess the association between T allele carriers and T2DM risk, with the CC genotype serving as the reference. * Statistically significant difference (*p* < 0.05) between CT + TT and CC genotypes in T2DM vs. control group, determined by Fisher’s exact test. *p*-values are uncorrected.

**Table 3:** Adjusted odds ratios and 95% confidence intervals for the association of type 2 diabetes mellitus with *transcription factor 7-like 2.* (rs7903146) genotype frequencies.

Parameter	Controls (*n* = 511)	T2DM (*n* = 600)	AOR ^a^	*p*-Value ^a^	95% CI
*TCF7L2* genotypes					
Dominant model					
CC genotype ^b^	496 (97.1)	568 (94.7)	1.00	-	-
CT/TT genotype	15 (2.9)	32 (5.3)	2.24	0.025	1.10–4.80
Alleles					
C allele (%)	1007 (98.5)	1167 (97.3)	0.20	0.016	0.05–0.75
T allele (%)	15 (1.5)	33 (2.8)	2.22	0.016	1.16–4.46

^a^: *p*-values are adjusted for age, sex, systolic blood pressure, diastolic blood pressure, body mass index, and smoking. ^b^: by logistic regression analysis to observe the risk of T variant in type 2 diabetes and used CC genotype as a referent group.

**Table 4:** Clinical and biochemical features of the study groups stratified by transcription factor 7-like 2 (rs7903146) genotypes.

	All Subjects			Type 2 Diabetes			Controls		
Parameters	CC	CT + TT	*p*-Value	CC	CT + TT	*p*-Value	CC	CT + TT	*p*-Value
N	1064	47		568	32		496	15	
Age	61.0 ± 16.2 (60.0–61.9)	65.5 ± 15.3 (61.0–70.0)	0.062	73.3 ± 6.5 (72.8–73.8)	74.0 ± 6.8 (71.5–76.5)	0.549	46.9 ± 12.0 (45.8–48.0)	47.4 ± 12.2 (40.6–54.2)	0.777
Sex (male/female)	531/533	22/25	0.678	280/288	15/17	0.857	251/245	7/8	0.799
BMI (male) (kg/m^2^)	25.6 ± 3.7 (25.4–25.9)	25.4 ± 3.5 (24.4–26.4)	0.732	26.0 ± 3.7 (25.7–26.3)	24.8 ± 3.5 (23.5–26.0)	0.384	25.3 ± 3.6 (25.0–25.6)	26.5 ± 3.3 (24.7–28.3)	0.251
BMI (female) (kg/m^2^)	24.5 ± 4.2 (24.2–24.8)	23.7 ± 4.7 (22.3–25.0)	0.329	24.0 ± 4.0 (23.8–24.3)	26.1 ± 3.7 (24.7–27.4)	0.024	22.7 ± 3.9 (22.4–23.0)	23.1 ± 6.3 (19.6–26.6)	0.716
WHR (male) (cm)	0.92 ± 0.07 (0.92–0.93)	0.92 ± 0.07 (0.90–0.94)	0.991	0.95 ± 0.06 (0.94–0.96)	0.93 ± 0.08 (0.90–0.96)	0.667	0.89 ± 0.07 (0.88–0.90)	0.90 ± 0.05 (0.87–0.92)	0.620
WHR (female) (cm)	0.85 ± 0.08 (0.84–0.85)	0.85 ± 0.06 (0.83–0.87)	0.655	0.89 ± 0.06 (0.88–0.90)	0.87 ± 0.04 (0.86–0.88)	0.132	0.79 ± 0.06 (0.78–0.80)	0.82 ± 0.08 (0.77–0.86)	0.470
SBP (mm Hg)	130 ± 19 (128–131)	132 ± 16 (127–137)	0.413	134 ± 19 (132–136)	132 ± 15 (126–137)	0.618	124 ± 18 (122–126)	131 ± 19 (120–142)	0.238
DBP (mm Hg)	77 ± 11 (76–78)	79 ± 13 (75–83)	0.351	78 ± 11 (77–79)	79 ± 12 (74–83)	0.633	77 ± 12 (75–78)	79 ± 15 (71–87)	0.370
TCHOL (mg/dL)	200.1 ± 40.7 (197.7–202.5)	194.1 ± 33.1 (184.4–203.8)	0.317	192.7 ± 40.4 (189.4–196.0)	195.0 ± 33.2 (183.0–206.9)	0.754	208.6 ± 39.3 (205.1–212.1)	192.2 ± 33.9 (173.4–210.9)	0.073
TG (mg/dl) *	107.0 (72.0–156.0)	114.0 (75.0–174.0)	0.206	122.5 (86.0–167.8)	118.5 (83.3–199.8)	0.220	92.0 (63.0–137.0)	100.0 (52.0–143.0)	0.958
HDL-C (mg/dL)	55.7 ± 17.0 (54.7–56.7)	54.0 ± 15.2 (49.5–58.5)	0.501	50.8 ± 13.2 (49.7–51.9)	51.8 ± 15.0 (46.3–57.2)	0.680	61.2 ± 19.1 (59.5–62.9)	58.5 ± 15.2 (50.1–66.9)	0.670
LDL-C (mg/dL)	119.4 ± 37.5 (119.8–125.9)	110.7 ± 29.5 (102.0–119.4)	0.115	108.8 ± 35.3 (105.9–111.7)	107.3 ± 29.9 (96.5–118.0)	0.808	131.5 ± 36.2 (128.3–134.7)	117.9 ± 28.2 (102.2–133.5)	0.109
FPG (mg/dL)	122.9 ± 51.4 (119.8–125.9)	140.5 ± 54.8 (124.4–156.6)	0.023	150.9 ± 56.6 (146.2–155.6)	162.5 ± 54.2 (142.9–182.0)	0.111	90.8 ± 8.3 (90.0–91.5)	95.3 ± 10.3 (89.6–101.0)	0.043
HbA1c (%)	7.0 ± 2.1 (6.8–7.1)	7.4 ± 2.2 (6.8–8.0)	0.216	8.3 ± 2.0 (8.1–8.5)	8.3 ± 2.0 (7.6–9.0)	0.727	5.5 ± 0.4 (5.4–5.5)	5.5 ± 0.3 (5.3–5.6)	0.508
Cr (mg/dL)	0.9 ± 0.3 (0.8–0.9)	0.9 ± 0.4 (0.8–1.0)	0.784	1.0 ± 0.4 (0.9–1.0)	1.0 ± 0.4 (0.9–1.1)	0.588	0.8 ± 0.2 (0.7–0.8)	0.8 ± 0.2 (0.7–0.9)	0.527
Hs-CRP (mg/L) *	1.2 (0.5–3.5)	1.3 (0.5–5.6)	0.043	2.6 (0.8–7.0)	3.6 (0.7–10.6)	0.045	0.7 (0.4–1.9)	0.8 (0.4–3.3)	0.779

Data are expressed as means ± standard deviation (95% confidence interval) for normally distributed variables, median (interquartile range) for skewed variables, and number for categorical variables. *p*-values are uncorrected and calculated using independent *t*-tests for normally distributed variables, Mann–Whitney U tests for skewed variables, and χ^2^ tests for categorical variables, as appropriate. Abbreviations: BMI, body mass index; WHR, waist-to-hip ratio; SBP, systolic blood pressure; DBP, diastolic blood pressure; TCHOL, total cholesterol; TG, triglyceride HDL-C, high-density lipoprotein cholesterol; LDL-C, low-density lipoprotein cholesterol. FPG, fasting plasma glucose; Cr, creatinine; Hs-CRP, high-sensitivity C-reactive protein. * Significant difference was tested using log-transformed data.

## Data Availability

Data supporting the findings of this study are available from the corresponding author upon reasonable request.

## Supplementary Materials

The following supporting information can be downloaded at https://www.mdpi.com/article/10.3390/diagnostics15162110/s1, Table S1: Transcription factor 7-like 2 (rs7903146) genotype distributions in the two study groups.

## Abbreviations

The following abbreviations are used in this manuscript:

*TCF7L2*	Transcription factor 7-like 2
T2DM	Type 2 diabetes mellitus
BMI	Body mass index
WHR	Waist-to-hip ratio
SBP	Systolic blood pressure
DBP	Diastolic blood pressure
LDL-C	Low-density lipoprotein cholesterol
HDL-C	High-density lipoprotein cholesterol
Hs-CRP	High-sensitivity C-reactive protein
SNP	Single nucleotide polymorphism
OR	Odds ratio
CI	Confidence interval
AOR	Adjusted odds ratio
GLP-1	Glucagon-like peptide-1
HMG	High-mobility group
