# Supplementary material for: Association of TCF7L2 rs7903146 (C/T) Polymorphism with Type 2 Diabetes Mellitus in a Chinese Population: Clinical Characteristics and Ethnic Context
Source: Diagnostics (Basel). 2025 Aug 21;15(16):2110. doi: 10.3390/diagnostics15162110 (PMC12385235; doi:10.3390/diagnostics15162110)
Supplement: Supplementary file 1 [file diagnostics-15-02110-s001.zip › diagnostics-3766134-supplementary.pdf]

SUPPLEMENTARY DATA

Association of *TCF7L2* rs7903146 (C/T) Polymorphism with Type 2 Diabetes Mellitus: Clinical and Ethnic Perspectives in a Chinese Population

Contents

Supplementary Table S1. *Transcription factor 7-like 2* (rs7903146) genotype distributions in the two study groups……………………………………………...2

Table S1. *Transcription factor 7-like 2* (rs7903146) genotype distributions in the two study groups

Genotype	Controls	T2DM	Effect size	Type 2 diabetes mellitus											
	(n, %)	(n, %)		WFH (n, %)	WOFH[a] (n, %)	Effect size	ADO≥60 (n, %)	ADO<60 (n, %)	Effect size	BMI≥27 kg/m^2 (n, %)	BMI<27kg/ m^2 (n, %)	Effect size	Male (n, %)	Female (n, %)	Effect size
N (%)	511	600		224(62.2)	136(37.8)		180(30.0)	420(70.0)		203(33.8)	397(66.2)		295(49.2)	305(50.8)	
CC	496(97.1)	568(94.7)	1.86	208(92.9)	128(94.1)	0.59	173(96.1)	395(94.1)	0.69	194(95.6)	374(94.2)	0.77	280(94.9)	288(94.4)	0.79
CT+TT	15(2.9)	32(5.3)*		16(7.1)	8(5.9)		7(3.9)	25(6.0)		9(4.4)	23(5.8)		15(5.1)	17(5.6)	
p-value			0.048			0.828			0.303			0.483			0.790

Abbreviations: WFH – with a family history of diabetes; WOFH – without a family history of diabetes; ADO – age at diabetes onset; BMI – body mass index; CI – confidence interval. a: 240 participants lacking family history data were excluded from subgroup analysis. b: Logistic regression was used to assess the association between T allele carriers and T2DM risk, with the CC genotype serving as the reference. *Statistically significant difference ($p < 0.05$) between CT+TT and CC genotypes in T2DM vs. control group, determined by Fisher's exact test.
